# Immuno-oncologic care during COVID-19: Challenges and opportunities for improving clinical care and investigation

**DOI:** 10.46439/cancerbiology.2.029

**Published:** 2021

**Authors:** Kristen Spencer, Eric A. Singer, Eugenia Girda

**Affiliations:** 1 Division of Medical Oncology, Rutgers Cancer Institute of New Jersey and Rutgers Robert Wood Johnson Medical School, New Brunswick, NJ, Unites States; 2 Section of Urologic Oncology, Rutgers Cancer Institute of New Jersey and Rutgers Robert Wood Johnson Medical School, New Brunswick, NJ, Unites States; 3 Division of Gynecologic Oncology, Rutgers Cancer Institute of New Jersey and Rutgers Robert Wood Johnson Medical School, New Brunswick, NJ, Unites States

**Keywords:** COVID-19, Cancer, Pandemic, Immunotherapy

## Abstract

Cancer care has been greatly impacted during the COVID-19 pandemic. The number of cases and deaths caused by the COVID-19 pandemic continues to escalate throughout the United States and the world. Worldwide, over 150 million people have been diagnosed with the coronavirus and more than 3 million have died. Now that we have gained additional experience with COVID-19, we are starting to learn its full impact on oncology care and its effects on the practice of medicine and clinical research.

## Introduction

Several studies have demonstrated that patients with cancer have worse outcomes when infected with COVID-19. Worldwide, over 150 million people have been diagnosed with the coronavirus and more than 3 million have died [1]. The overlap between clinical characteristics of patients affected by cancer and those affected by COVID-19 make it challenging to discern risk factors between these populations. Advanced age, presence of medical comorbidities such as metabolic syndrome, morbid obesity, chronic cardiac and pulmonary disease, smoking, and immunosuppression have been risk factors common to both cancer and COVID-19 [2,3]. Earlier data from China demonstrated that patients with cancer are at higher risk for severe events that require intensive care unit (ICU) admission, ventilation support, or result in death compared with patients without cancer [4]. Other retrospective cohort studies demonstrated that 30-day all-cause mortality was higher among patients with cancer [5]. Patients who have undergone recent surgery [6], and those with hematologic cancer, lung cancer, or metastatic cancer appear to be particularly at risk [7]. Interestingly, even a remote history of cancer multiplied the risk of severe outcomes, possibly due to protracted immunodeficiency [8]. Together, these findings suggest that patients with cancer are significantly more vulnerable to the SARS-CoV-2 virus, and the COVID-19 pandemic will continue to pose challenges to oncology care.

Several cancer societies have issued guidelines to help manage oncology patients in the setting of COVID-19 [9–11]. Oncologists also independently started to employ several strategies to mitigate risks of exposure in cancer patients. Adjuvant chemotherapy and surgery for stable cancers were delayed in endemic areas [12,13]. Concerns regarding chemotherapy-induced immunosuppression led to modifications of chemotherapy regimens [14]. Frequency of treatment visits was decreased, and there was increased utilization of telehealth consultations and remote laboratory visits to reduce patients’ encounters with healthcare facilities [15]. However, in some patients, treatment may not be feasibly delayed, while certain populations such as the elderly, socioeconomically disadvantaged, and non-English speaking may be at a disadvantage when it comes to access to and utilization of these modern approaches to health care provision [16, 17]. Lastly, the unique mechanism of action and side effect profile of immune-oncology (IO) drugs pose unique challenges for oncology providers. As such, these topics warrant further consideration.

In addition to examining special circumstances in the provision of oncologic care during the COVID-19 pandemic, in this commentary we examine the unique challenges surrounding the use of IO drugs in susceptible patients. Special attention must be given to identifying and differentiating the symptoms of IO-related adverse events from COVID infection. The opportunities and challenges of expanded telemedicine services are discussed, including its impact on access to care and clinical research.

## Use of Immune-Based Therapies

A growing number of cancer patients are currently receiving or will be considered for various immunotherapies to manage their disease. Published reports on cancer patients with COVID-19 infection reflect this, with approximately 4–6% of patients receiving immunotherapy in the month prior to COVID-19 symptom onset [5,7]. Thus, a particular area of early research interest became the interplay between COVID-19 and patients receiving IO agents. One area of focus has been the susceptibility to and severity of infection from COVID-19 in patients receiving IO agents. A study at Memorial Sloan Kettering Cancer Center reported an association of checkpoint inhibitor immunotherapy as a risk factor for severe COVID-19 outcomes, independent of age, cancer type, and other comorbid conditions [18]. However, several other studies demonstrated that recent use of immunotherapy did not increase the risk of mortality in patients with COVID-19 [19,20]. While the numbers are small and cofounding factors exist, there is rationale to support the concern that patients receiving IO agents may have an enhanced immune system response to infection [5,21]. While initiation and progression of cancer occurs in the context of a suppressed immune response [22], the augmented immune response such as that which occurs in the setting of IO-based therapy can be an important predisposing factor for severe illness in patients with COVID-19 [5,23], perhaps placing these patients more at risk for life-threatening infection. These conflicting data complicate decision making with regard to what may be commonly perceived as less immunosuppressive systemic therapy alternatives.

## Differentiating IO Adverse Events from COVID-19 Symptoms

The overlap of well characterized immune-mediated toxicities and symptoms of COVID-19 infection presents an additional challenge of clinical relevance. By now it is known that symptoms of SARS-CoV-2 infection range from none or minimal manifestations to respiratory failure and severe cytokine storm [21]. Uniquely, as opposed to other coronavirus syndromes, death from COVID-19 is more commonly due to multi-organ failure rather than respiratory failure alone [4,24–26]. Compounded with the indiscriminate nature of ICI toxicities, multiorgan vulnerability to COVID-19 emphasizes the need for timely but accurate diagnosis and appropriate management of symptoms equally likely to be related to viral infection as to complications of treatment. Differentiating between the two is critical for several reasons, including the prompt initiation of non-steroid based COVID-19 supportive care measures that may be potentially lifesaving, the initiation of appropriate later line therapies for refractory ICI-related toxicities that could prove fatal otherwise, and more practically for guidance on further use of IO agents and eligibility for clinical trial participation.

Examined in more detail, the most common presenting symptoms of COVID-19 infection include fever (64–100%), cough (25–82%), dyspnea (25–44%), and fatigue or malaise (43%) [5,18,27], arguably indistinct from those seen with immune-mediated pneumonitis [28]. Further complicating the picture is that traditional manifestations of IO toxicity not initially felt to be overlapping features of COVID-19 are now felt to be common, potentially due to the constitutive expression of angiotensin-converting enzyme (ACE2), the functional receptor for the SARS-CoV-2 virus, on epithelial cells in the lung, kidney and intestine [29]. For example, one report described diarrhea as a presenting symptom in 26% of COVID-19 positive patients and demonstrated that presentation with acute onset diarrhea was predictive of need for hospitalization and severe illness due to COVID-19 in cancer patients receiving ICI therapy [18]. Likewise, nephritic syndrome, which has previously been described as the most common acute renal injury in the setting of ICI use [30], has been identified as a prognostic feature in patients with COVID-19 [31] and described at autopsy in COVID-19 patients [32]. Traditional radiography is also non-diagnostic, often with variable but overlapping presentations such as bilateral ground glass opacities with or without consolidation consistent with a cryptogenic organizing pneumonia common in both COVID-19 and immune-mediated pneumonitis [28], and enterocolitis reported in both COVID-19 [33] and ICI-induced colitis. While diagnostic criteria to aide in the diagnosis and management of both COVID-19 infection and immune-mediated toxicities are well developed, limited recommendations exist for the approach to patients on ICI therapy who develop clinical or radiographic findings suggestive of both etiologies. Herein we propose general recommendations that may be applied in this context. These guidelines are not meant to supplant management deemed clinically appropriate on a case-by-case basis with multidisciplinary consultation as indicated.

We propose asymptomatic patients with radiographic findings suggestive of COVID-19, or mildly symptomatic patients may be evaluated via telemedicine while self-isolating at home, with recommendations for supportive symptom management. In both situations, and particularly in those with radiographic suggestion of COVID-19 where chest CT abnormalities may be identified prior to the manifestation of symptoms [34], we recommend offering minimal contact testing for SARS-CoV-2 if available regionally. These patients should be monitored frequently for the development of and/or worsening of symptoms through the generally accepted incubation period, with in-person evaluation if indicated. Additionally, we recommend holding ICI therapy until the patient has remained stable without symptoms through the incubation period, or until resolution of mild symptoms.

In situations where immune-mediated toxicity cannot be reasonably differentiated from COVID-19 infection, and/or in patients with more than mild symptoms, in-person evaluation is warranted. We recommend this be done in a clinically appropriate setting in a dedicated COVID-19 assessment area with adequate infection prevention measures in place, including private rooms with negative-pressure ventilation and appropriate personal protective equipment (PPE) to include a face mask (potentially a fit-tested N95 mask), gloves, gown, and protective eyewear. Patients who report such symptoms at the time of a routine monitoring visit should be promptly isolated and evaluated in a similar fashion. If such procedures are not feasible, we recommend patients be referred to the nearest hospital for evaluation.

The initial evaluation of symptoms in patients with overlapping potentially immune-mediated and COVID-19 symptoms should begin broadly, including evaluations for any appropriate alternate etiology in parallel to evaluation for COVID-19 and ICI-toxicity. In cancer patients in particular, other infectious and thrombotic etiologies and disease progression should remain high on the differential. In addition to a focused physical exam, if radiographic imaging is necessary, CT scans are preferred given their superior diagnostic capabilities in the setting of both SARS-CoV-2 infection and immune-mediated toxicity [35,36]. We recommend considering the incorporation of laboratory tests such as ferritin, erythrocyte sedimentation rate (ESR), C-reactive protein (CRP), lactate dehydrogenase (LDH), D-dimer, and interleukin (IL)-6 into the initial evaluation as, while these studies are not yet validated in this setting, they may help differentiate between potential diagnoses, and they are often being incorporated into the workup of COVID-19 in clinical practice [37]. Diagnostic procedures, though often recommended as part of the initial workup of suspected immune-mediated pathologies, should be avoided unless absolutely indicated due to the risk of viral transmission and potential for deterioration in clinical status.

The role of corticosteroids in the management of COVID-19 patients presents another conundrum in patients receiving CPI therapy, where steroids are often the mainstay of symptom management. Initial reports suggested the use of corticosteroids in patients with COVID-19 resulted in higher utilization of ICU-level care and death due to severe infection [38–40]. The more recent RECOVERY trial demonstrated the use of dexamethasone decreased 28-day mortality in patients receiving respiratory support (invasive or non-invasive), however there was no evidence for a benefit in patients not receiving respiratory support and may have been harmful in these patients [41]. A subsequent meta-analysis confirmed these findings [42]. Contrary to the primary approach to severe ICI-toxicities, high doses of steroids may be harmful in the initial stages of infection where viral shedding is at a peak, but immunopathological mechanisms play a less predominant role [41].

As such, patients with confirmed COVID-19 should be routinely assessed for indications to use corticosteroids, however their empiric use in patients being evaluated for both COVID-19 and ICI toxicity should be considered cautiously based on symptom grade. Avoiding steroids in lieu of symptomatic management in patients with suspected grade one ICI-toxicity can be considered. For patients with grade 2 symptoms, where steroids would typically be initiated empirically for suspected ICI toxicity, holding their use until after confirmation of COVID-19 status may be considered. For patients with grade 3 or higher symptoms, we continue to recommend the use of empiric steroids in parallel with COVID-19 testing and discontinuation of steroids in the setting of confirmed COVID-19 infection, unless indicated by severity of infection or concomitant immune-medicated toxicity is suspected. Additionally, anti-IL-6 agents (e.g. tocilizumab, sarilumab) in the event of steroid-refractory symptoms should be considered, as these therapies have been efficacious in controlling both steroid-refractory immune-mediated toxicities [43] and severe COVID-19 infection [44–47]. As more data becomes available, we advocate for the incorporation of anti-IL-6 therapy as the initial therapy for patients with symptoms potentially due to COVID-19 or ICI-toxicity given their shared mechanism of action described above. See Table 1 for a summary of recommendations.

**Figure 1:**
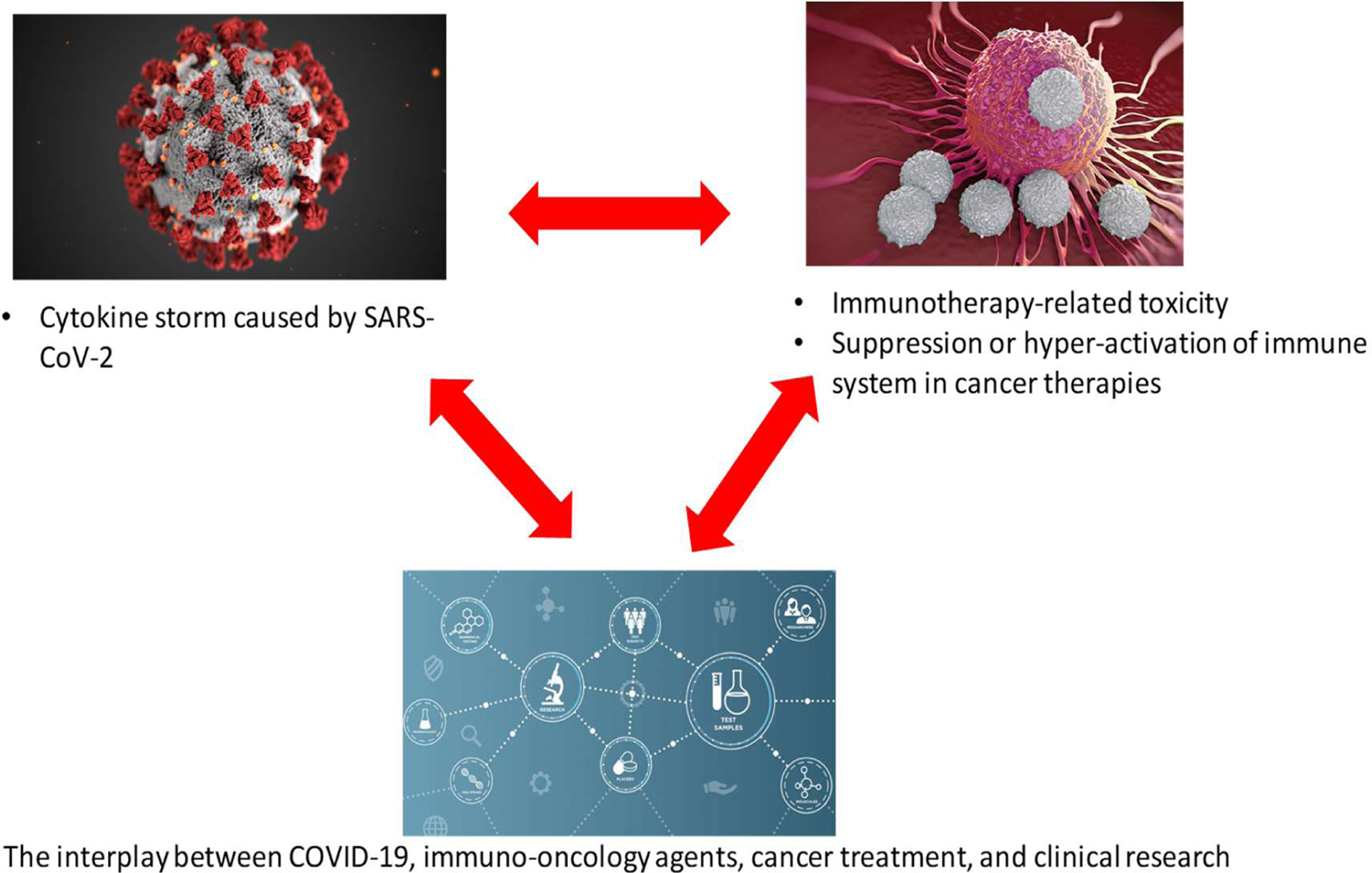
Immuno-Oncologic Care During COVID-19: Challenges and Opportunities for Improving Clinical Care and Investigation.

Lastly, prevention of COVID-19 infection in patients on immunotherapy-based treatments deserves special consideration. Current guidelines recommend new longer interval ICI dosing schedules to minimize exposures to the healthcare system [48], however patients transitioning to less frequent dosing regimens may be at particular risk due to higher Cmin and Cmax levels of the drug early after treatment initiation [49], and a potentially corresponding proliferative immune system response that subsequently dissipates over time. Patients initiating IO-based therapy are at similar risk [21]. Similarly, consideration should be given to cessation of ICIs in patients who have received complete responses or have been on therapy at least two years as has been done in melanoma and non-small cell lung cancer studies respectively. Careful attention should be paid to the prevention of co-infection through both standard prophylactic vaccination and appropriate opportunistic infection coverage in patients requiring long-term immunosuppression for management of toxicities. Finally, ICI use should be used with caution in patients with risk factors concomitant with severe COVID-19 infection (e.g., elderly, current smokers, comorbidities such as diabetes or COPD) [48] (Figure 1).

## Access to Care

The COVID -19 pandemic fundamentally changed the practice of oncology, not only by altering the types of cancer therapies, but also by shifting the mode of cancer delivery. Telemedicine has rapidly moved to the forefront of clinical practice in response to the COVID-19 pandemic. As suggested above, telemedicine does allow for fewer encounters between patients and the healthcare system in general, a known risk factor for COVID-19 infection. However, we must consider whether there are particular subsets of patients in whom, or particular situations in which, a virtual visit may not be an adequate replacement for an in-person evaluation. By way of example, we suggest considering in-person evaluations for all patients initiating IO therapy or transitioning to a new IO administration schedule. Additionally, we prioritize in-person evaluation of symptoms that require a physical assessment that may not be adequately achieved by a telemedicine visit, especially in the subsets of patients particularly pre-disposed to severe COVID-19 infection, such as patients with lung cancer or hematologic malignancies [11]. Patients with lung cancer are particularly complex as ICI therapy is frequently used, and symptoms related to the disease may overlap with symptoms of both immune-mediated pneumonitis and COVID-19.

Un-insured and under-insured patients represent a subset of patients rarely considered in recommendations for the routine use of telemedicine in place of standard care practices. This has become particularly salient in light of the fact that over 40 million Americans filed for unemployment during the period of mid-March to late May 2020 at the peak of the COVID pandemic alone. A survey from the Commonwealth Fund reported 41% of those who lost a job or whose spouse lost a job relied on that job for insurance, and 20% of these Americans did not have alternate insurance coverage [50]. This adds to the disproportionate numbers of Black or African American, Asian, and Hispanic/Latino patients who are un- or under-insured according to the U.S. Bureau of Labor Statistics. The significance of this is difficult to ignore, as inadequately insured patients seek medical care less often, conceivably including less prophylactic COVID testing and vaccination, and under-assessment and treatment of symptomatic illness to minimize disease severity and spread. Additionally, un- and under-insured patients with chronic comorbidities, particularly those with immunosuppressive effects, are less likely to have baseline control of their conditions making them even more susceptible to severe disease.

Lastly, practitioners routinely face challenges in providing care virtually to patients functionally unable to utilize them. This includes socioeconomically disadvantaged patients who lack the technology to participate in telemedicine visits, elderly patients who so often do not have an understanding of, or assistance with the technology, and non-English speaking patients who may have difficulty with the communication required to initiate a virtual visit. Table 2 summarizes unique challenges to modern care provision among special populations and provides recommendations to overcome such challenges.

## Impact on Clinical Research

Finally, substantial changes in our clinical research practices in order to adjust to the pandemic may have led to significant setbacks for our research patient population that will have lasting effects on the future of cancer therapy. Many clinical trials were suspended to protect patients and staff from the risk of infection and to shift resources to expedite clinical research investigating treatments and a vaccine for COVID-19. Both the U.S. Food and Drug administration (FDA) and the National Cancer Institute (NCI) released guidelines for physicians caring for and recruiting patients to research studies and the companies sponsoring those trials, often resulting in rapid changes to protocols. The FDA acknowledged and allowed for protocol modifications to address challenges that may arise during the conduct of clinical trials. These have included, but are not limited to, unexpected quarantines or adverse events related to COVID-19 exposure or infection, study site closures, travel limitations, and interruption of supply chains, all of which may lead to difficulty in meeting protocol-specific procedures such as protocol-mandated visits or laboratory/diagnostic testing. See Table 3 for a summary of considerations and modifications.

The emergence of COVID-19 vaccines introduced another level of uncertainty for oncology patients on chemotherapy, immunotherapy, and clinical trials. With lack of safety and efficacy data in the cancer patient population, more information is required in this subset of patients to answer questions about the time needed for immunity to develop, duration of such immunity, the effects of a variety of systemic treatments on immunity, and optimal timing of vaccine administration [51]. However, given the greater severity of the COVID-19 infection and higher risk of death in patients with cancer, as highlighted above, multiple organizations recommended prioritizing COVID-19 vaccinations for patients with cancer [52,53]. Patients treated with investigational agents on clinical trials are encouraged to receive the COVID-19 vaccine, further highlighting the focus on patient safety. Initial controversies surrounding COVID-19 vaccinations in the setting of clinical trials were around the fact that in many countries, current vaccines are “authorized” rather than “approved”, making them investigational agents, which are usually not allowed concurrently on a clinical trial. However, the FDA provided operational guidance for clinical trial sponsors, investigators and treating physicians, clarifying that a COVID-19 vaccine is not considered an investigational agent. Therefore, we recommend offering immunization against COVID-19 to all patients participating on clinical trials if clinically appropriate, though the safety and efficacy questions mentioned above remain of particular interest.

## Conclusions

COVID-19 continues to disrupt healthcare systems. Cancer patients remain especially vulnerable given the toxicities associated with their treatments and the need for frequent visits with their healthcare providers. Patients receiving IO drugs may need additional support and counseling to differentiate irAEs from possible COVID symptoms. Oncology practices, both academic and private, can take concrete steps to minimize patient risk while expanding access to care and facilitating the continuity of clinical investigation. Ongoing research into best practices and planning for spikes in COVID cases remain critical. While the COVID-19 pandemic undoubtedly places significant strain on cancer care delivery system, it is prudent that we use our experience and expertise thus far to improve the quality and resiliency of this system.

## Figures and Tables

**Table 1:** Summary of Initial Recommendations for Management of Overlapping Immunotherapy Toxicity and COVID-19 Symptoms in Patients with Unknown COVID-19 Status.

Scenario	Management Considerations
Asymptomatic patient with concerning radiographic findings	Hold immunotherapy (resume after incubation period if remains asymptomatic) Work-up as clinically indicated to include COVID-19 testing Self-isolation Minimal contact testing (where available) Supportive care with symptom management Symptom follow up through incubation period
Mildly symptomatic patients	Hold immunotherapy (resume after resolution of symptoms according to CDC guidelines) Work-up as clinically indicated to include COVID-19 testing Self-isolation Minimal contact testing (where available) Supportive care with symptom management Symptom follow up through incubation period
Suspected grade 1 immunotherapy toxicity	Hold immunotherapy (resume after resolution of symptoms according to CDC guidelines) Work-up as clinically indicated to include COVID-19 testing Avoid corticosteroid use Supportive care with symptom management
Suspected grade 2 immunotherapy toxicity	Hold immunotherapy (resume after resolution of symptoms if clinically indicated according to ASCO and CDC guidelines) Work-up as clinically indicated to include COVID-19 testing Supportive care with symptom management
Suspected grade 3 or higher immunotherapy toxicity	Hold immunotherapy (resume after resolution of symptoms if clinically indicated according to ASCO and CDC guidelines) Work-up as clinically indicated to include COVID-19 testing Empiric corticosteroids per institutional standards Consider anti-IL-6 therapy up front or if steroid-refractory

**Table 2:** Recommendations for Management of the Evolving Healthcare Landscape.

Challenge	Considerations
Determining appropriateness for telemedicine encounter	Consider avoiding telemedicine visits for particular subsets of patients: Patients with symptoms overlapping between COVID or immune-mediated toxicity Patients whose symptoms require in-person assessment for adequate evaluation Patients initiating on immunotherapy Patients transitioning to new immunotherapy dosing schedule
Resource access	Expand access to free or low-cost in-person assessments for symptom management, as well as routine care of comorbid conditions Free access to COVID testing Free access to vaccine (when available) Patient assistance programs to provide low cost previously owned devices (e.g., tablets, cellular telephones) to access visits Outreach to public entities (e.g., libraries) to provide access to same devices on-site or on-loan to access visits Dedicated information technology (IT) services at institutions with access to live support for connection and technology issues
Special populations	Outreach to communities disproportionately unemployed and un- or under-insured (e.g., Black or African American, Asian, and Hispanic/Latino communities) to facilitate connection with healthcare coverage Outreach to volunteer organizations to assist elderly patients with access to and utilization of devices to access visits Development of technology to incorporate translation services
Supporting telemedicine encounters	Pre-screening of patients before encounters to ensure accuracy of medical information Pre-screening of patients before encounters to address additional needs that will likely come up during the encounter (e.g., financial, emotional, palliative, logistical) Dedicated ancillary services to rapidly address needs uncovered during encounter
Policy	Re-consideration of reimbursement policies that are based on telemedicine format given limitations addressed herein

**Table 3:** Conduct of Clinical Research during COVID-19 Pandemic.

Limitations	Considerations
Safety of trial participants	Interruption of investigational drug for patients already on trial Change in patient monitoring schedule Telehealth and virtual research visits Use of electronic signatures for patient consent forms Suspension of trial recruitment (if indicated)
Laboratory/Imaging tests	Alternative (local) location for tumor imaging assessment and labs Flexible timelines for required laboratory tests or imaging
Site closures/interruption of supply chain	Shipping of oral medications directly to patients
Monitoring visits	Utilizing central or remote monitoring programs to maintain oversight of clinical sites
Delay in research progress	Extending budget period for approved projects
